# OTME-19. Regula Regulation of Gliomagenesis and Stemness Through Acid Sensor ASIC1a

**DOI:** 10.1093/noajnl/vdab070.070

**Published:** 2021-07-05

**Authors:** Pendelton King, Jingwei Wan, Alyssa guo, Shanchun Guo, Yugang Jia, Mingli Liu

**Affiliations:** 1 Morehouse School of Medicine, Atlanta, GA, USA; 2 The Second Xiangya Hospital, Central South University, Changsha, Hunan, China; 3 University of South Carolina SOM Greenville, Greenville, SC, USA; 4 Xavier University, New Orleans, LA, USA

## Abstract

Glioblastoma multiforme (GBM) is the most prevalent and aggressive type of adult gliomas. Despite intensive therapy including surgery, radiation, and chemotherapy, invariable tumor recurrence occurs, which suggests that glioblastoma stem cells (GSC) render these tumors persistent. Recently, GSC differentiation has emerged as an alternative method to treat GBM, and most of current studies aim to convert GSC to neurons by a combination of transcriptional factors. As the tumor microenvironment is typically acidic due to increased glycolysis in tumor cells, here, we explored the role of acid-sensing ion channel 1a (ASIC1a), an acid sensor, as a tumor suppressor in gliomagenesis and stemness. The bioinformatics data from TCGA shows that ASIC1 expression levels in GBM tumor tissues were lower than those in normal brain, and glioma patients with elevated ASIC1 expression have longer survival than those with lowered ASIC1 expression. Our immunohistochemistry data from tissue microarray shows that ASIC1a expression is negatively correlated with glioma grading. Functional studies reveal that the downregulation of ASIC1a promotes glioma cell proliferation and invasion, while upregulation of ASIC1a inhibits their proliferation and invasion. Furthermore, ASIC1a suppresses glioma cells’ growth and proliferation through G1/S arrest and apoptosis induction. Mechanistically, ASIC1a negatively modulates glioma stemness via inhibition of the Notch signaling pathway and GSC markers CD133 and ALDH1. Our findings indicate that ASIC1a is a tumor suppressor in gliomagenesis and stemness and may serve as a promising prognostic biomarker and target for GBM patients.

